# Dental Students' Perception and Self-Perceived Confidence Level in Key Dental Procedures for General Practice and the Impact of Competency Implementation on Their Confidence Level, Part I (Prosthodontics and Conservative Dentistry)

**DOI:** 10.1155/2023/2015331

**Published:** 2023-10-11

**Authors:** Wijdan R. Elmanaseer, Salah A. Al-Omoush, Rasha A. Alamoush, Rawan Abu Zaghlan, Firas Alsoleihat

**Affiliations:** ^1^Department of Prosthodontics, School of Dentistry, The University of Jordan, Amman 11942, Jordan; ^2^Department of Restorative Dentistry, School of Dentistry, The University of Jordan, Amman 11942, Jordan

## Abstract

**Background:**

Evaluating the level of dental students' competence is crucial for validating their preparedness for graduation. Confidence has a significant role in achieving competence. There are limited studies that assess the level of self-perceived confidence among final-year dental students regarding their ability to conduct key dental procedures. This study aims to assess the self-perceived confidence level of final-year dental students in performing essential dental procedures across various dental disciplines and to assess the effect of implementing competencies in the curriculum on the self-perceived confidence level of students by comparing two cohorts of final-year students in two different years 2016 (Traditional Cohort) and 2019 (Competencies Cohort).

**Materials and Methods:**

An questionnaire was answered by two cohorts of final-year dental students: one group in 2016 before the implementation of the competency-based assessment system (group 1, *n* = 153), and the other in 2019 after the implementation of this system (group 2, *n* = 199), the same questionnaire was used for both cohorts. The results from the two groups were compared regarding the degree of self-perceived confidence in conducting key dental procedures. The data were analysed using SPSS statistics and Levene's Test for Equality of Variances and *t*-test for Equality of Means calculated.

**Results:**

Group 1 showed a significantly higher means of self-perceived confidence levels than group 2 in the ability to conduct seven out of the 20 prosthodontics procedures studied: providing patients with Cobalt–Chromium (Co–Cr) removable partial dentures (RPD) (3.77 vs. 3.56), providing the patient with Acrylic RPD (3.70 vs. 3.23), treatment planning for partially edentulous patients (3.83 vs. 3.34), giving OHIs for denture patients (4.17 vs. 3.95), dealing with CD postinsertion complaints (3.97 vs. 3.76), giving postinsertion instructions for removable prostheses cases (4.12 vs. 3.82), and providing patients with immediate dentures (2.67 vs. 2.32). The same applies to 6 out of 16 conservative dentistry procedures: placing anterior composite (4.41 vs. 4.12), placing posterior composite (4.43 vs. 3.88), placing posterior amalgam (4.29 vs. 4.02), placing matrix band for Class II restorations (4.24 vs. 3.71), placing a prefabricated post (3.34 vs. 2.88), and placing fiber post (3.45 vs. 3.34). On the other hand, group 2 shows higher means of self-perceived confidence than group 1 in only two conservative dentistry procedures: onlay restorations (2.18 vs. 2.76) and inlay restorations (2.22 vs. 2.75). No significant differences in means of self-perceived confidence were found between the two groups in the remaining 21 procedures studied.

**Conclusions:**

This study has shown that final-year dental students have high self-perceived confidence levels in doing simple dental procedures yet less confidence in more complex ones. Although, students' self-perceived confidence decreases after the introduction of a competency-based assessment system. Competency implementation and execution criteria may differ between schools which may have an impact on final outcomes. Hence, there is a need for regular evaluation of competencies being assessed to maintain a curriculum that is up to date.

## 1. Introduction

During their education journey, dental students classically progress through progressive phases of education starting with theory, moving onto preclinical and paraclinical training, and ending with clinical education. This progression aims to ensure that students develop the necessary knowledge and expertise to become dentists with sufficient capabilities to practice safely in their careers [1]. Throughout the educational process assessment of students is crucial to assess the degree of achievement of the intended learning objectives and the effectiveness of the education provided. Effective education requires teaching and assessment strategies to be aligned with the intended learning outcomes which are usually grounded on the necessities of clinical reality [2]. Competency is the ability to combine evidence-based knowledge, personal attitudes, and clinical skills to undertake holistic dental care [3, 4]. Competency may be of greater relevance to dental practice than confidence; however, the role of confidence in achieving competence should not be underestimated [5]. Quality of education and clinical experience are integral to determining the competence and self-perceived confidence level of dental students [6]. Hence, the implementation of competencies in the curricula of undergraduate dental students was to support them to develop the capacity to become safe practitioners, and as a tool to update and develop curricula [7–11].

Internationally, competency profiles may have slight differences between different dental schools; however, they all share common core competencies that are designed to ensure both independent and safe clinical practice [12]. These shared competencies normally consist of the ability to deal with clinical and scientific knowledge, to communicate, to have appropriate social skills, to diagnose and to make a treatment plan, to take care of the patient, to promote health and prevention, and to have a professional attitude [13]. As mentioned earlier, the need to evaluate the level of dental students' competence is crucial for validating their preparedness for graduation. With the continuous introduction of new scientific knowledge, the descriptors for the level of competencies are continuously questioned, thus the need to constantly restructure the requirements required to obtain competencies. Nowadays, new clinical challenges not encountered at dental school are encountered by recent graduates. They are often required to perform invasive or noninvasive and often nonreversible surgical procedures using sophisticated materials and equipment. Therefore, it is of great importance to encourage educational methods that allow graduates to cope with unforeseeable developments [14]. This exerts more stress on the graduating dentists, as they are expected not only to be attentive to patients' needs, but also to learn the new skills required to treat them appropriately [15]. It is important to assess the self-perceived confidence level of dental students especially those about to graduate regarding key dental procedures.

In addition to the quality of education, clinical experience plays an essential role in determining the self-perceived confidence level of dental students [6]. Although studies have revealed varying results, some have shown that dental students lacked confidence when performing complicated dental procedures [16]. However, this self-perceived confidence level increased when students gained more experience and training [17].

In the past few years, some dental institutions along with other human science schools have encountered many challenges including the increased number of enrolled students exceeding the planned capacity of the schools [18]. In addition, the limited availability of patients negatively affected the level of clinical training for the students [19]. These challenges have stretched our dental institution to sustain the intended level of education and training for its students. Dental school of the University of Jordan is not far from the previously mentioned challenges, dental students need 5 years of successful continuous studying to graduate, they have preclinical laboratory training in year 3, then in year 4 they start their clinical training; however, most clinical requirements and competencies are in their final (fifth) year.

There is limited information regarding the way students perceive competencies and their self-perceived confidence level in various dental procedures. Few studies have been conducted in Jordan to assess the self-perceived confidence levels of dental students regarding various dental procedures which are needed by new graduates to practice as general practitioners [20]. No studies have compared students' self-perceived confidence levels over time.

Prosthodontics and conservative dentistry are core disciplines in dental practice. General dental practitioners encounter both simple and complicated cases after graduation. Consequently, general dental practitioners should have the ability to evaluate and diagnose properly and to perform, to a satisfactory standard, many procedures, especially for simple cases. This paper assesses prosthodontic and conservative dental procedures, while a subsequent paper will consider other procedures. It is a comparative study between 2019 where students have to achieve the required competencies to pass the course and 2016 where competencies were not included in the curriculum.

In 2017, competencies were introduced for the first time in the curriculum of the dental school at the University of Jordan. This was because, currently the methods of education are shifting from traditional to new interactive problem-based learning (PBL) which includes group discussions, case-based learning, and self-assessment approaches. Competency-based education is part of the new educational methods which aim in improving their self-perceived confidence and experience. Competencies involve multiple tasks that students are able to try to fulfill independently without supervision, they were either pass or fail and need to be achieved to enter the final clinical exam at the end of the final (fifth) year. It was hoped that they would encourage students to undertake dental procedures with more confidence. Competencies are part of the fourth and fifth years, but most of the requirements and competencies are in the final year. Therefore, fifth-year students were chosen for this study. The only difference between the two cohorts is the addition of competencies (requirements of each year stayed the same), the questions of the questionnaire were based on the list of competencies provided by each department to each course.

Surveys are an appropriate technique to evaluate students' perceptions and to gather information in a way that allows educators, to address the successfulness and the limitations of the educational experience [21–23]. This study aims to assess the self-perceived confidence level of final-year dental students in performing essential dental procedures from core dental disciplines; prosthodontics and conservative dentistry. Additionally, to assess the effect of implementing competencies in the curriculum on the self-perceived confidence level of students by comparing two cohorts of final-year students in two different years 2016 (Traditional Cohort) and 2019 (Competencies Cohort).

## 2. Materials and Methods

### 2.1. Data Collection

This study was carried out in two stages; stage one was conducted at the end of the academic year of 2015/2016 when competencies were not part of the curriculum. All fifth-year dental graduates (*n* = 153) were asked to complete a paper-based questionnaire. Stage two was conducted at the end of the academic year 2019/2020 when competencies were part of the curriculum. The same questionnaire, but in an online format (Google Forms), was sent to all fifth-year dental graduates and they were asked to fill it (*n* = 199), online questionnaire was used in the second stage for the ease of data processing. The only significant change that took place in the curriculum and assessment methods of the two studied groups was the implementation of the competency-based system, and no multiple interventions took place to influence the results. In addition, the staffing was consistent throughout. The requirements for both cohorts stayed the same, competencies were implemented in each course of the 2019 cohort as part of the requirements needed except they were assessed on pass-or-fail bases. Competencies were required to be successfully fulfilled by students to be allowed to enter the final exam; however, in both cohorts, the students were required to finish a set of requirements to pass the course.

The sample size was estimated using G. power 3.03 using a high effect size of 0.2, at a power of 0.95 at 0.05 one-tailed level of significance using Pearson correlation as test statistics for one sample. This estimation showed that the sample size needed for the study is 146 participants.

The questionnaire items were based on all the required competencies that are included in the curriculum from the core dental specialties (prosthodontics, conservative dentistry, endodontics, pediatrics, oral surgery, orthodontics, periodontics, and radiology) in the University of Jordan. Accordingly, the questionnaire was divided into eight sections, each section was related to the chosen discipline as the following, prosthodontics (20 questions), conservative dentistry (16 questions), endodontics (nine questions), radiology (four questions), pediatrics (12 questions), orthodontics (seven questions), oral surgery (15 questions), and periodontics (nine questions). The questions were designed to assess the students' self-perceived confidence level in completing the clinical tasks. The responses were reported on a five-point scale Likert scale from (strongly confident, confident, neutral, not confident, and strongly not confident). The questionnaire was validated and found reliable prior to being dispatched to the participants. The questionnaire was piloted on a 5% sample of the group (final-year students) to test the instrument's psychometric properties and discover difficulties that might be encountered during the actual data collection, and check the tools' convenience to Jordanian culture. The pilot study also helped determine the time needed for the participants to complete the questionnaire, its readability, and clarity.

### 2.2. Ethical Approval

This study was approved by the Academic Research Committee of the School of Dentistry/the University of Jordan (Ref. Number 9-2019). Before the students filled out the questionnaire, the questionnaire stated that the participants were not obliged to complete and return the forms and that completion of the survey would have no influence on their overall academic grading or performance. To maintain anonymity, no personal identifiers were used in the online questionnaire.

### 2.3. Data Analysis

Data were collected, coded, and screened for completeness before entering the computer program. The analysis was performed using Statistical Package for Social Science (SPSS) version 22. The distribution of the variables was reviewed for skewed distribution. The descriptive statistics recorded were frequencies, mean, median, mode, standard deviation, and percentages according to the level of variables. Inferential analysis assessed the impact of competencies implementation in curricula on the self-perceived confidence level of students. Levene's Test for Equality of Variances and *t*-test for Equality of Means were conducted to compare both samples and to insure normal distribution. The total mean of the self-perceived confidence level for each procedure included here it was compared between the two cohorts using the two-independent samples *t*-test, and the level of confidence was set at 95% level. Statistical significance was set at the 0.05 level.

### 2.4. Hypothesis

Introducing a competency-based assessment for final-year dental students at the University of Jordan is expected to enhance their self-perceived confidence levels in performing essential dental procedures.

## 3. Results

A total of 352 fifth-year dental students were included in the study (153 from 2016 and 199 from 2019) with a 100% response rate.

### 3.1. Students' Perception and Self-Perceived Confidence Level in Prosthodontic

**Figure 1:**
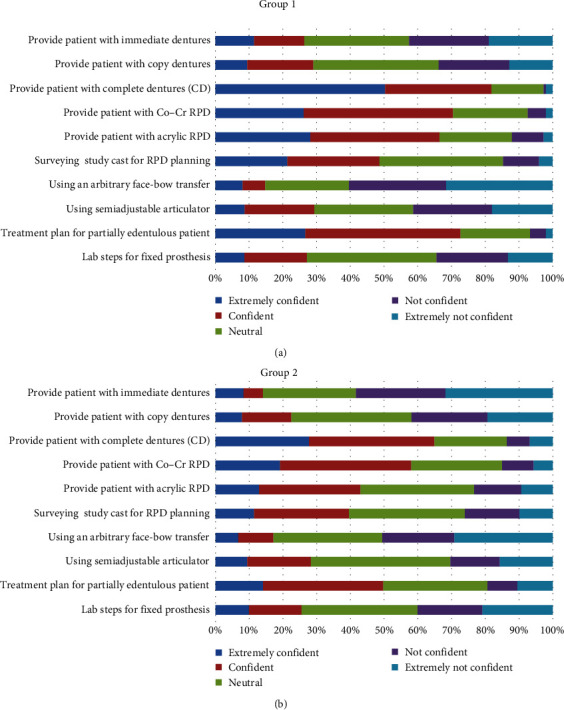
The level of confidence in the ability to conduct a number of key prosthodontic procedures among (a) group 1 and (b) group 2.

**Figure 2:**
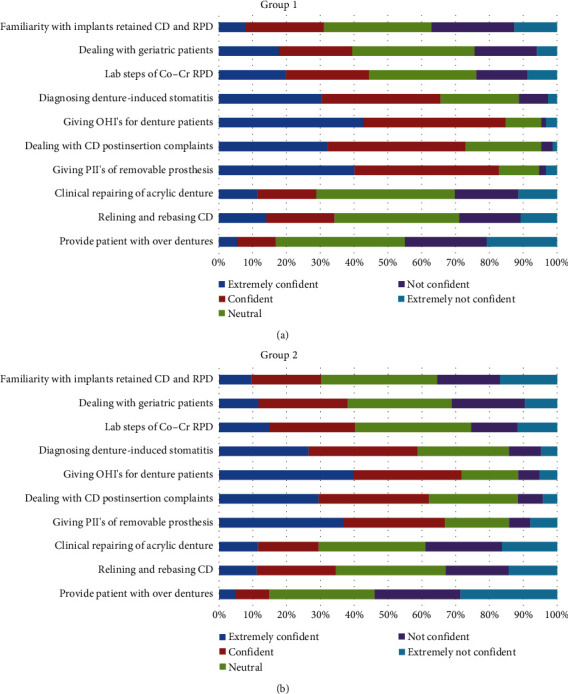
The level of confidence in the ability to conduct a number of key prosthodontic procedures among (a) group 1 and (b) group 2.

In group 1, the majority of students reported being “strongly confident” or “confident” in eight of the 20 procedures: providing patients with CD (72%), Cr–Co (70%), and acrylic dentures (66%); treatment planning for the partially edentulous patients (73%); diagnosis of denture stomatitis (65%); giving oral hygiene instructions (OHI's) (85%); dealing with CD postinsertion complaints (73%); and giving postinsertion instructions (PII's) for removable prosthesis (83%). For only one procedure, using a face-bow, most students reported being “not confident” or “strongly not confident” (61%). In group 2, the majority of students reported being “strongly confident” or “confident” in the same procedures as group 1, except in one procedure which is providing patients with acrylic partial dentures (43%). On the other hand, most students were “not confident” or “strongly not confident” in providing immediate dentures (43% and 59%), using an arbitrary face-bow (61% and 50%), and providing overdentures (45% and 54%) (Figures 1 and 2).

Comparing both groups, the mean of self-perceived confidence significantly dropped (*P* < 0.05) in seven out of 20 prosthodontics procedures after the implementation of the competency-based system: providing patients with Cobalt–Chromium (Co–Cr) removable partial dentures (RPD) (3.77 vs. 3.56), providing the patient with Acrylic RPD ((3.70 vs. 3.23), treatment planning for partially edentulous patients (3.83 vs. 3.34), giving OHIs for denture patients (4.17 vs. 3.95), dealing with CD postinsertion complaints (3.97 vs. 3.76), giving postinsertion instructions for removable prostheses cases (4.12 vs. 3.82), and providing patients with immediate dentures (2.67 vs. 2.32) (Table 1).

No significant difference in confidence was found between the two groups in the rest of the procedures, and for no procedure did the confidence increase in the competency cohort.

### 3.2. Students' Perception and Self-Perceived Confidence Level in Conservative Dentistry

**Figure 3:**
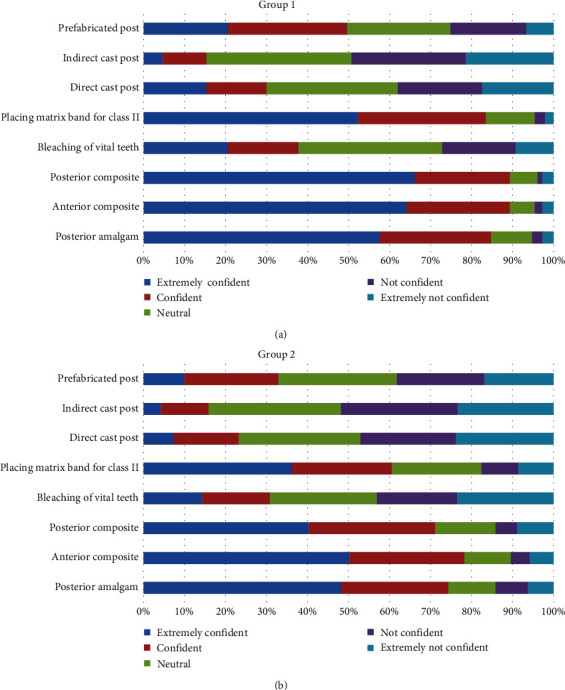
The level of confidence in the ability to conduct a number of key conservative procedures among (a) group 1 and (b) group 2.

**Figure 4:**
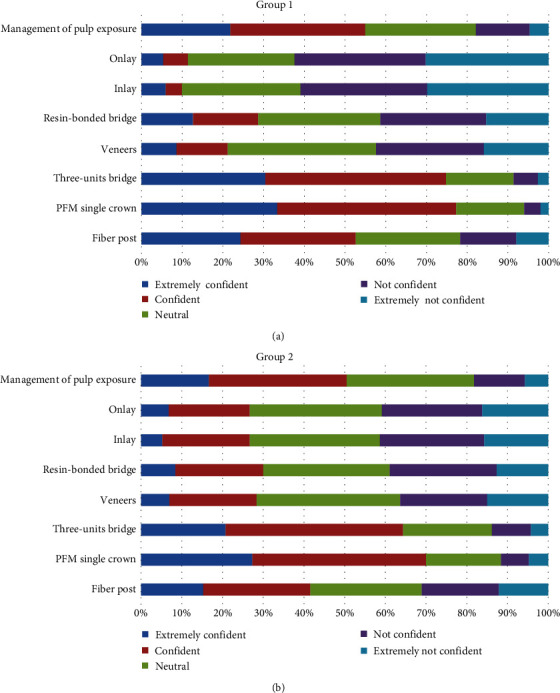
The level of confidence in the ability to conduct a number of key conservative procedures among (a) group 1 and (b) group 2.

In group 1, the majority of students reported being “strongly confident” or “confident” in eight of the 16 procedures: placement of anterior and posterior composite fillings (89% and 89%), placement of posterior amalgam fillings (85%), placement of matrix band for class II fillings (83%), placement of prefabricated post and fiber post (50%, 52%), management of iatrogenic pulp exposure (55%), providing patients with porcelain fused to metal (PFM) single crowns (77%) and three-units bridge (74%). But the majority of students reported being “strongly not confident” or “not confident” in only two procedures: providing inlays and onlays (61% and 62%), and providing indirect posts (49%). In group 2, as in group 1, most students reported being “strongly confident” or “confident” in the same procedures except in the placement of prefabricated posts (33%). However, students were not confident in providing indirect cast posts only (52%) (Figures 3 and 4).

The results showed that the degree of confidence (total mean) significantly dropped (*P* < 0.05) in six out of 16 conservative dentistry procedures after the implementation of the competency-based system: placing anterior composite (4.41 vs. 4.12), placing posterior composite (4.43 vs. 3.88), placing posterior amalgam (4.29 vs. 4.02), placing matrix band for class II restorations (4.24 vs. 3.71), placing a prefabricated post (3.34 vs. 2.88), and placing fiber post (3.45 vs. 3.34) (Table 2).

No significant difference in confidence was found between the two groups in the rest of the procedures, although the confidence significantly increased (*P* < 0.05) in six out of 16 procedures after the implementation of the competency-based system: onlay restorations (2.18 vs. 2.76) and inlay restorations (2.22 vs. 2.75) (Table 2).

### 3.3. Fi-Index Tool

This manuscript has been checked with the Fi-index tool and obtained a score of 0.88 for the first author only on the date 20/02/2023 according to SCOPUS® [24, 25]. The fi-index tool aims to ensure the quality of the reference list and limit any autocitations.

## 4. Discussion

Continuous revision and development of curriculum and assessment methods hold great significance in dental education. Comparing different assessment methods, researchers can determine which ones are more effective in evaluating a student's performance accurately and consistently [26]. Studies comparing assessment methods in dental education play a vital role in improving teaching and learning, curriculum development, and the overall quality of dental education. This in turn ensures that students are adequately confident and prepared for their future roles as dental professionals [27]. In this study, students showed high self-perceived confidence levels in doing simple procedures in both cohorts (traditional or competencies cohorts), for example, providing patients with simple removable prostheses, treatment planning for partially edentulous patients, placement of amalgam and composite fillings, single crown PFM and three-units bridge. This is similar to the results reported in previous studies [5, 23, 28, 29]. Murray showed that in final-year students from New Zealand that 68.4% of students were highly confident/confident in providing patients with acrylic RPD, 59.6% in providing full CD, 84.5% and 77.6% in providing anterior and posterior composite restoration, respectively, 47.4% in conventional bridge preparation, 87.8% in crown preparation [30].

Similarly, the low level of self-perceived confidence which was commonly reported for more complex procedures (immediate or overdentures, implant retained prosthesis, inlay, onlay, veneers, and resin-bonded bridges) is reported in other studies. In another recent study carried out in Jordan, 97% of the fifth-year students felt extremely confident in doing direct restorations while their self-perceived confidence level was significantly lower in doing indirect restorations [20]. This could be related to the concept of a “safe beginner” who acts within the boundaries of their own capabilities and limitations and knows when to request support and advice, although it has been suggested that this definition lacks both precision and detail [31]. Postgraduate experience and training in more complex procedures should increase self-perceived confidence levels as clinical training and experience are one of the main factors that affect self-perceived confidence levels [5, 6, 32]. Restricting participants to one stage of clinical experience could be considered a limitation of this study and could be addressed in future research by investigating the confidence of dentists after their internship period.

The results of the present study do not agree with the assumption that competency assessment might have a positive effect on the self-perceived confidence level of students, as this enhancement is only demonstrated in two out of 36 procedures investigated. The finding that for all prosthodontics procedures self-perceived confidence levels reduced or stayed constant is initially counterintuitive. However, it may be that the students start to focus on the competency tests rather than more holistically, a good example of assessment driving learning. A similar conclusion can be reached for the conservative procedures, where the only increase in confidence was for more complex procedures where, perhaps, students had less experience prior to the introduction of mandatory competency assessments. Basically, competencies designed by professionals are needed skills that represent the bases in curriculum development, student assessment, and accreditation. Similarly, the concept of competency-based education has been suggested to improve critical thinking and autonomy while embracing knowledge and confidence as well [28]. However, due to intrinsic institutional constraints, the comprehensive approach in dental education cannot always be practiced in its entirety or perfectly. At the time of the study, our school curriculum could be described as a hybrid one, since it included competencies in all dental disciplines as mentioned before alongside the needed requirements. Moreover, achieving self-confidence is an important asset in enhancing competency [33], but to avoid students being overconfident they need to learn how to self-evaluate their performance. This ability to self-evaluate and achieve the real confidence needed is important because it is directly affecting the results of studies that measure the self-perceived confidence level, in a recent study, final-year dental students appear to have high self-confidence in basic areas of general dentistry, but when compared to summative assessment, confidence appears to be overestimated [34]. Self-confidence is best achieved by independent clinical practice and implementing reliable methods for evaluating competency during undergraduate training; Kaufman et al. [35] stated that learners should be able to analyze and assess their own performance and develop new perspectives and options. Another recent study compared dental graduates of two universities concluded that high self-perceived confidence levels could be related to more clinical practice in the specialty during undergraduate trainin [36]. Consequently, the self-perceived confidence between the two cohorts in this study was not in favor of the competencies' positive effect. Furthermore, the results in this study may be explained by the methods used for the evaluation of the competencies and by the set-up of competencies besides the requirements needed from fifth-year students to successfully graduate. Competencies can be evaluated by traditional or more modern methods [37]. The methods used for evaluation in our dental school are traditional methods, where the students must complete a series of competencies during their clinical training, the task must be done independently in a specific period, and the task is subjectively evaluated by two assessors. In addition, the competency assessment includes short oral questions about the procedure which aim to assess knowledge. This system converts each task into a “high-stakes” assessment—where there is a good evidence suggesting that candidates do not perform to their usual standard. This may, in part, contribute to the overall finding of lower overall confidence amongst the 2019/20 cohort. Furthermore, the traditional method had a major drawback in that it is subjective and occasionally inflexible, while newer criteria look to objectively at assessing the students according to set standards or criteria. Consequently, the current method for competency assessment needs to be revised to successfully assess the intended learning outcomes of the curriculum.

## 5. Conclusions

Competency implementation and execution criteria may differ between schools which may have an impact on final outcomes. Hence, there is a need for regular evaluation of competencies being assessed to maintain a curriculum that is up to date. In addition, a regular evaluation of assessment methods to ensure that they maintain fitness for purpose as the curriculum changes are required.

## Figures and Tables

**Table 1:** Comparison of the level of confidence in the ability to conduct key prosthodontic procedures between group 1 and group 2.

Procedure	Year	Mean	*SD*
Lab steps for fixed prosthesis	2016	2.84	1.165
	2019	2.74	1.233

Treatment plan for partially edentulous patient	2016	3.83	1.056
	2019	3.34	1.149

Using semiadjustable articulator	2016	2.73	1.262
	2019	2.92	1.158

Using an arbitrary face-bow transfer	2016	2.25	1.253
	2019	2.44	1.205

Surveying study cast for RPD planning	2016	3.44	1.164
	2019	3.15	1.132

Provide patient with acrylic RPD	2016	3.70	1.193
	2019	3.23	1.133

Provide patient with Co–Cr RPD	2016	3.77	1.109
	2019	3.56	1.079

Provide patient with complete dentures (CD)	2016	4.16	1.115
	2019	3.72	1.143

Provide patient with copy dentures	2016	2.82	1.236
	2019	2.69	1.172

Provide patient with immediate dentures	2016	2.67	1.322
	2019	2.32	1.215

Provide patient with over dentures	2016	2.50	1.165
	2019	2.37	1.139

Relining and rebasing CD	2016	3.01	1.259
	2019	2.98	1.204

Clinical repairing of acrylic denture	2016	2.91	1.216
	2019	2.86	1.228

Giving PII's of removable prosthesis	2016	4.12	0.993
	2019	3.82	1.223

Dealing with CD postinsertion complaints	2016	3.97	0.949
	2019	3.76	1.086

Giving OHI's for denture patients	2016	4.17	0.979
	2019	3.95	1.137

Diagnosing denture induced stomatitis	2016	3.77	1.127
	2019	3.66	1.112

Lab steps of Co–Cr RPD	2016	3.27	1.253
	2019	3.18	1.194

Dealing with geriatric patients	2016	3.25	1.160
	2019	3.09	1.152

Familiarity with implants retained CD and RPD	2016	2.86	1.178
	2019	2.87	1.201

RPD, removable partial dentures.

**Table 2:** Comparison of the level of confidence in the ability to conduct key conservative dentistry procedures between group 1 and group 2.

Procedure	Year	Mean	*SD*
Posterior amalgam	2016	4.29	1.068
	2019	4.02	1.218

Anterior composite	2016	4.41	1.029
	2019	4.12	1.143

Posterior composite	2016	4.43	1.018
	2019	3.88	1.247

Bleaching of vital teeth	2016	3.18	1.273
	2019	2.79	1.355

Placing matrix band for class II	2016	4.24	1.037
	2019	3.71	1.277

Direct cast post	2016	2.84	1.338
	2019	2.60	1.219

Indirect cast post	2016	2.44	1.129
	2019	2.45	1.098

Prefabricated post	2016	3.34	1.247
	2019	2.88	1.227

Fiber post	2016	3.45	1.251
	2019	3.34	3.072

PFM single crown	2016	3.95	1.069
	2019	3.81	1.062

Three-units bridge	2016	3.89	1.067
	2019	3.67	1.043

Veneers	2016	2.68	1.173
	2019	2.84	1.134

Resin-bonded bridge	2016	2.79	1.286
	2019	2.87	1.145

Inlay	2016	2.22	1.131
	2019	2.75	1.119

Onlay	2016	2.18	1.155
	2019	2.76	1.148

Management of pulp exposure	2016	3.50	1.176
	2019	3.43	1.086

PFM, porcelain fused to metal.

## Data Availability

The datasets used and/or analyzed during this study are available on reasonable request from the corresponding author.
